# IL-27, but not IL-35, inhibits neuroinflammation through modulating GM-CSF expression

**DOI:** 10.1038/s41598-017-16702-w

**Published:** 2017-11-29

**Authors:** Giacomo Casella, Annamaria Finardi, Hélène Descamps, Federico Colombo, Chiara Maiorino, Francesca Ruffini, Marco Patrone, Massimo Degano, Gianvito Martino, Luca Muzio, Burkhard Becher, Roberto Furlan

**Affiliations:** 1 Clinical Neuroimmunology Unit, Department of Neuroscience, Institute for Experimental Neurology, San Raffaele Scientific Institute, 20132 Milan, Italy; 2 Neuroimmunology Unit, Department of Neuroscience, Institute for Experimental Neurology, San Raffaele Scientific Institute, 20132 Milan, Italy; 3 Biocrystallography Unit, Department of Immunology, Transplantation and Infectious Diseases, San Raffaele Scientific Institute, 20132 Milano, Italy; 4 Inflammation Research, Institute of Experimental Immunology, University of Zurich, 8057 Zurich, Switzerland

## Abstract

IL-27 and IL-35 are heterodimeric cytokines, members of the IL-12 family and considered to have immunomodulatory properties. Their role during neuroinflammation had been investigated using mutant mice devoid of either one of their subunits or lacking components of their receptors, yielding conflicting results. We sought to understand the therapeutic potential of IL-27 and IL-35 delivered by gene therapy in neuroinflammation. We constructed lentiviral vectors expressing IL-27 and IL-35 from a single polypeptide chain, and we validated *in vitro* their biological activity. We injected IL-27 and IL-35-expressing lentiviral vectors into the cerebrospinal fluid (CSF) of mice affected by experimental neuroinflammation (EAE), and performed clinical, neuropathological and immunological analyses. Both cytokines interfere with neuroinflammation, but only IL-27 significantly modulates disease development, both clinically and neuropathologically. IL-27 protects from autoimmune inflammation by inhibiting granulocyte macrophages colony-stimulating factor (GM-CSF) expression in CD4^+^ T cells and by inducing program death-ligand 1 (PD-L1) expression in both CNS-resident and CNS-infiltrating myeloid cells. We demonstrate here that IL-27 holds therapeutic potential during neuroinflammation and that IL-27 inhibits GM-CSF and induces pd-l1 mRNA *in vivo*.

## Introduction

The interleukin-12 family of heterodimeric cytokines is characterized by three alpha subunits (p19, p28, p35) and two beta subunits (p40 and EBI3), allowing six theoretical different pairings^1,2^. While for IL-12 (p35/p40), IL-23 (p19/p40) and IL-27 (p28/EBI3) there is concluding evidence that the pairing occurs *in vivo*, the existence and biological relevance of IL-35 (p35/EBI3), IL-39 (p10/EBI3), and IL-Y (p28/p40) are questioned by some authors^1^. Structure homology within the IL-12 family leads, surprisingly, to very different biological properties. In general, IL-12 and IL-23 are considered pro-inflammatory cytokines whereas IL-27 and IL-35 are mainly anti-inflammatory or immunoregulatory cytokines^2^.

IL-27 is a pleiotropic cytokine mainly released by antigen-presenting cells (APC), modulating several immune cells, including myeloid cells and T cells^3^. IL-27 signals through a heterodimer composed by IL-27R alpha (also called WSX-1) and gp130, that can be found on both innate- and acquired-immune cells and that induces STAT1/STAT3 phosphorylation^4,5^. IL-27, initially thought to be a pro-inflammatory, Th1-promoting, cytokine^6^, suppresses Th1, Th17 responses and limits CNS inflammation in several experimental models^7^. In fact, in experimental autoimmune encephalomyelitis (EAE), recombinant IL-27 peripheral administration inhibits disease development^8^, while interference with IL-27 signaling results in exaggerated Th17 responses and the worsening of EAE^8,9^. IL-27 has been shown to inhibit the development of Th17 cells and to promote the differentiation of IL-10 producing type 1 regulatory T cells (Tr1 cells)^8,10^. The suppressive effect of IL-27 on GM-CSF, a cytokine considered crucial for the development of autoimmune neuroinflammation^11,12^, has been suggested *in vivo* using deletion mutants for one of the subunits composing either the cytokine or its receptor^8,11^. However, direct evidence of IL-27 inhibitory activity on GM-CSF in neuroinflammation is lacking.

IL-35 is an immune-regulatory cytokine, produced *in vitro* by mouse and human Foxp3^+^ Treg cells and B cells^13–16^. IL-35 signaling has been described to occur either through IL-12Rβ2/gp130, gp130/gp130, or WSX1/IL-12Rβ2, inducing phosphorylation of STAT1/STAT4 or STAT1/STAT3^17^. IL-35 can suppress T cell proliferation by inducing cell-cycle arrest in G1 phase without inducing apoptosis^15,18^. Very little is known on the role of IL-35 in EAE, mostly through indirect genetic models yielding contradictory results. EBI3-deficient mice, for example, lacking both IL-27 and IL-35, display slightly increased severity of EAE on one hand, but produce more potent EAE-suppressive Tregs on the other hand^19^. Deletion of p35 selectively in B cells causes inability of mice to recover from EAE^16^.

To gain direct information on the role of IL-27 and IL-35 during EAE we engineered lentiviral vectors to express IL-27 and IL-35 from a single polypeptide chain, and delivered them directly in the CNS of EAE mice. We found that CNS expression of IL-27, but not IL-35, inhibits EAE development, possibly by modulating GM-CSF and pd-l1.

## Results

### IL-27 and IL-35 lentiviral vectors express functional proteins

**Figure 1:**
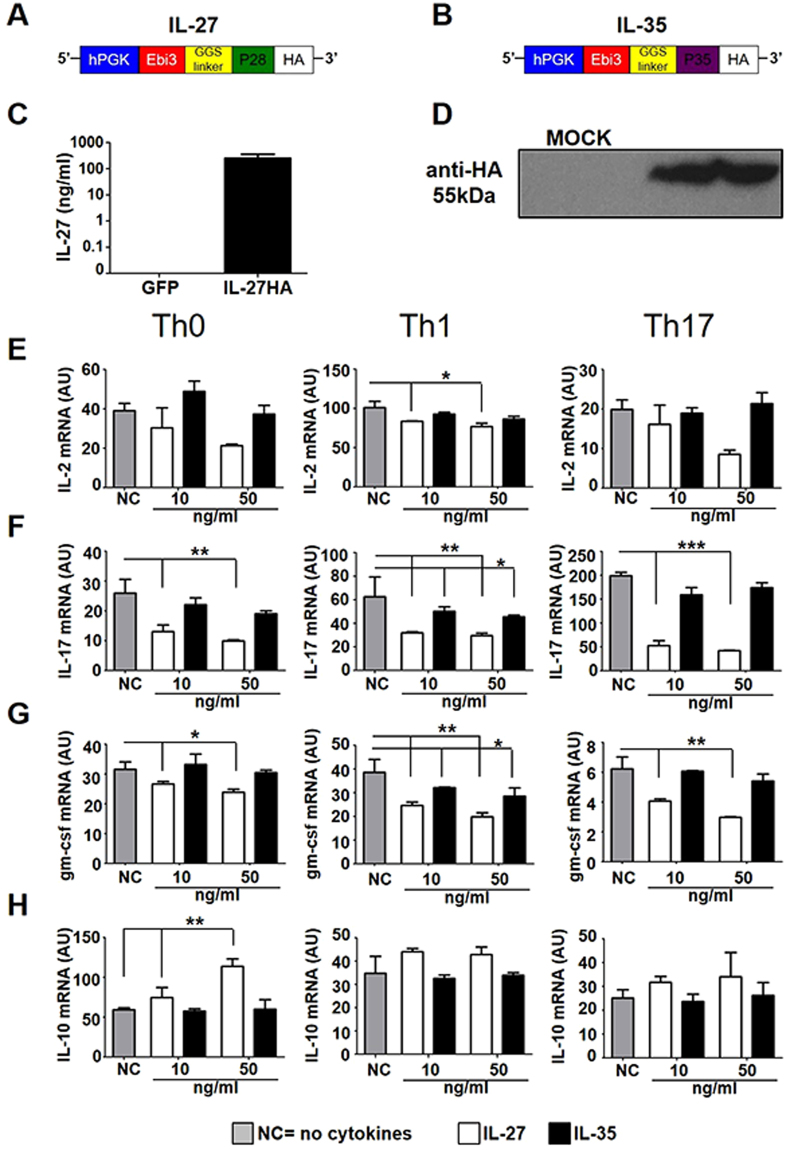
Construction and validation of Lenti-IL-27HA and Lenti-IL-35HA. (**A**,**B**). Graphic representation of the lentiviral constructs used to express IL-27 (**A**) and IL-35 (**B**). (**C**,**D**) Cytokines production was assessed by ELISA (IL-27, **C**) or WB (IL-35, **D**). (**E**–**G**). Affinity purified IL-27 (white bars) and IL-35 (black bars) were used to treat polarized CD4^+^ T cells and measure mRNA expression of IL-2 (**E**), IL-17 (**F**), gm-csf (**G**), and IL-10 (**F**) (mean ± sd). NC = no cytokines. *p < 0.05, **p < 0.001, ***p < 0.0001 (1 way Anova). Blot in panel (**D**) has been cropped, and full blot is presented in Supplementary Fig. 7. We show here one representative out of three independent experiments.

Using lentiviruses, we expressed the heterodimeric cytokines IL-27 (Lenti-IL-27HA) and IL-35 (Lenti-IL-35HA) from a single polypeptide chain, inserting a GGS linker long and flexible enough to allow the correct pairing of the two subunits, and an HA tag at the C-terminus (Fig. 1A,B). We used a GFP-expressing lentivirus as control (Lenti-GFP). We checked IL-27 and IL-35 by ELISA and WB (Fig. 1C,D) and we administered the purified proteins at different concentrations, 10 ng and 50 ng, to CD4^+^ T cells polarized *in vitro* towards the Th0, Th1 and Th17 phenotype (Supplementary Fig. 1A–D). IL-27HA and IL-35HA did not change viability or proliferation of CD4^+^ T cells (Supplementary Fig. 1E–G). IL-27HA inhibited, in a dose-dependent way the expression of IL-2, IL-17 and gm-csf both at the mRNA (Fig. 1E–G) and protein level (Supplementary Fig. 2A–C), while IL-35HA was less consistent, confirming however, its reported ability to inhibit the release of IL-2, IFNγ and TNFα from stimulated CD4^+^ Th1 and Th17 cells (Fig. 1F–G; Supplementary Fig. 2A,E,F). Moreover, IL-27 but not IL-35 induced IL-10 mRNA (Fig. 1H) and protein (Supplementary Fig. 2D) in CD4^+^ T cells. Our cytokines, IL-27HA and IL35HA, work very similar to available recombinant IL-27 and IL-35 (Supplementary Figs 3 and 4). These data support the idea that IL-27HA and IL-35HA drive the expression of proteins with biological functions similar to recombinant IL-27 and IL-35.

### Intrathecal injection of Lenti-GFP, Lenti-IL27HA, and Lenti-IL35HA in EAE mice drives the release of GFP, IL-27, and IL-35 in the CSF

**Figure 2:**
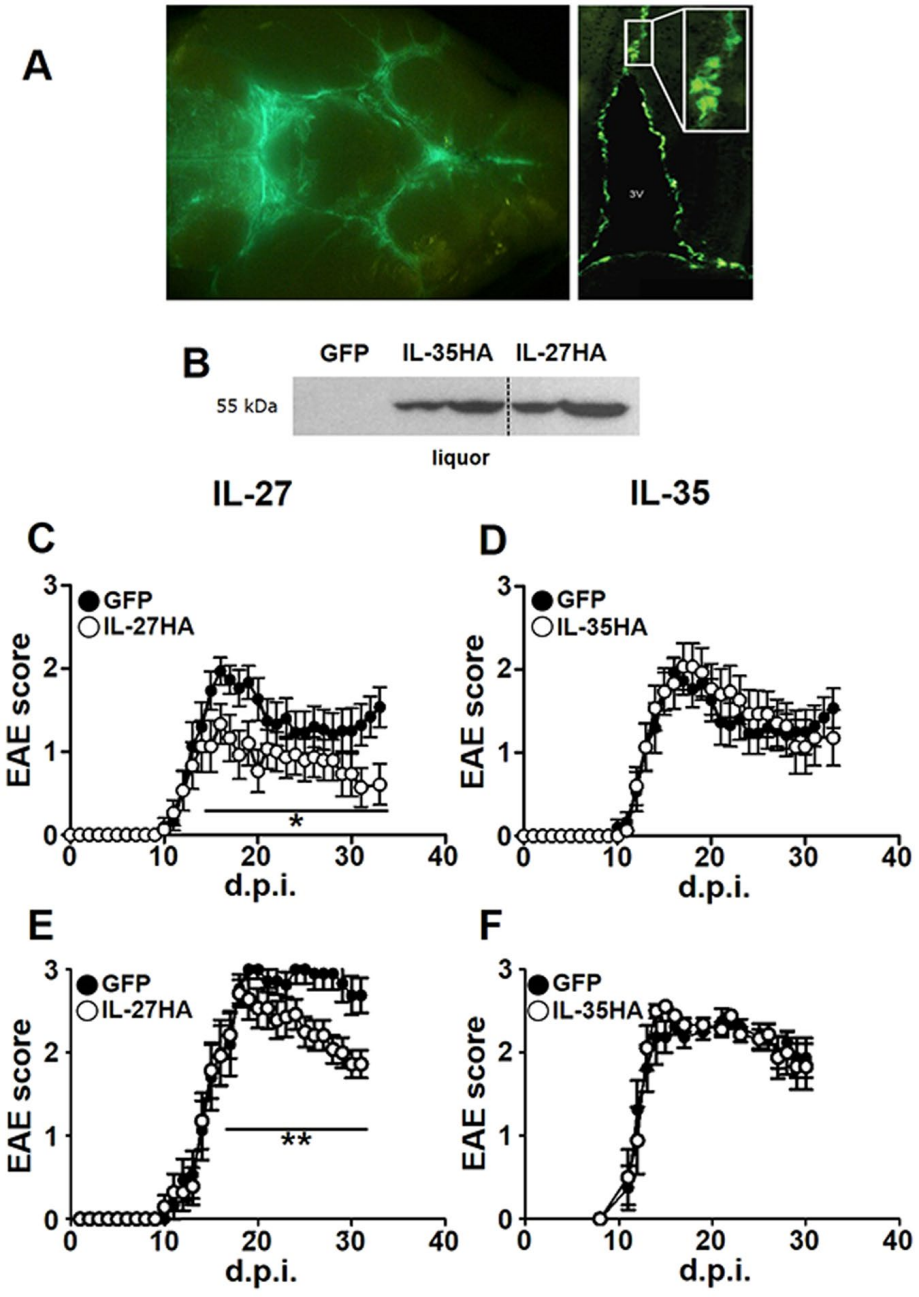
IL-27, but not IL-35 gene therapy inhibits clinical EAE development. Intracisternally injected lentiviruses infected leptomeningeal (**A**, right panel) and ependymal (**A**, inset) cells releasing transgenes product into the CSF (**A**), and as shown by WB (dotted vertical line) are stitching of adjacent pictures (**B**). Mean clinical score of EAE mice injected with Lenti-IL-27HA and Lenti-IL-35HA (open dots) or Lenti-GFP (closed dots) both preventively (**C,D**), on the day after immunization, and therapeutically, on the day of disease onset (**E**,**F**). *p < 0.05; **p < 0.001 (EAE is evaluated as cumulative score using Mann Whitney; n = 15/group). We show here one representative out of three independent experiments (see Supplementary Figure 5 for the other two EAE experiments). Mice were sacrificed between 28–34 days post immunization. Blot in panel (**B**) has been cropped, and full blot is presented in Supplementary Fig. 7.

We injected Lenti-IL-27HA, Lenti-IL-35HA, and Lenti-GFP into the *cisterna magna* of EAE mice, as previously described^20^. We obtained efficient infection of leptomeningeal (Fig. 2A, left panel) and ependymal (Fig. 2A, right panels) cells, and we confirmed the release, in comparable amounts, of the expected cytokines in the CSF by WB using the anti-HA antibody (Fig. 2B).

### IL-27, but not IL-35, gene therapy protects from neuroinflammation

**Figure 3:**
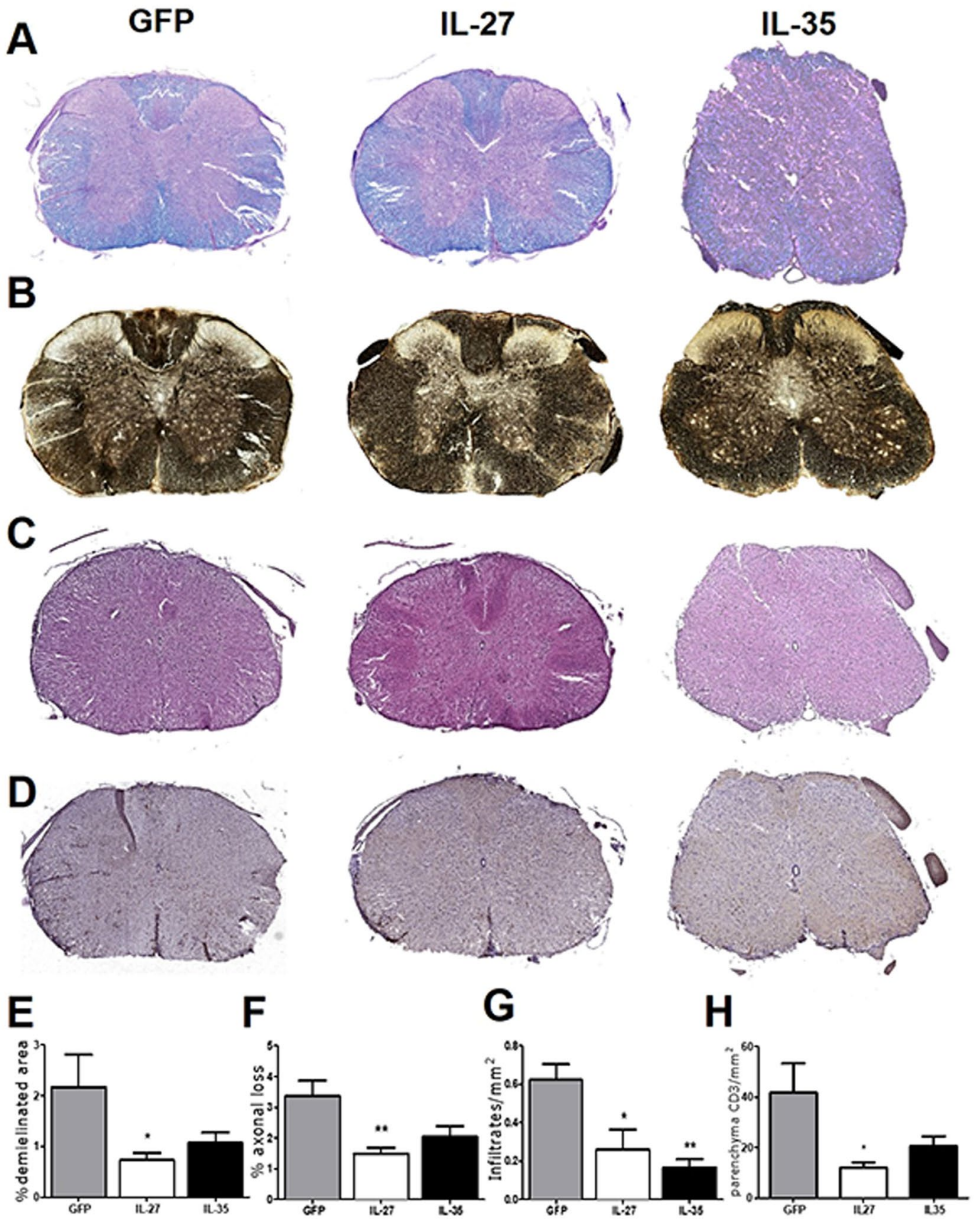
Intrathecal IL-27, and IL-35 gene therapy protects EAE mice from tissue damage. Neuropathological analysis of demyelination (**A**), axonal loss (**B**), infiltrates (**C**), and CD3^+^ T cells (**D**). The protective effect of IL-27HA gene therapy is associated to a significant decrease of demyelination (**E**), axonal damage (**F**), inflammatory infiltrates (**G**), and number of infiltrating CD3^+^ T cells (**H**) (mean ± sd). IL-35HA gene therapy promoted a significant decrease of inflammatory infiltrates (**G**). *p < 0.05; (1 way anova; **E–H** n = 5/group). We show here one representative out of three independent experiments.

**Figure 4:**
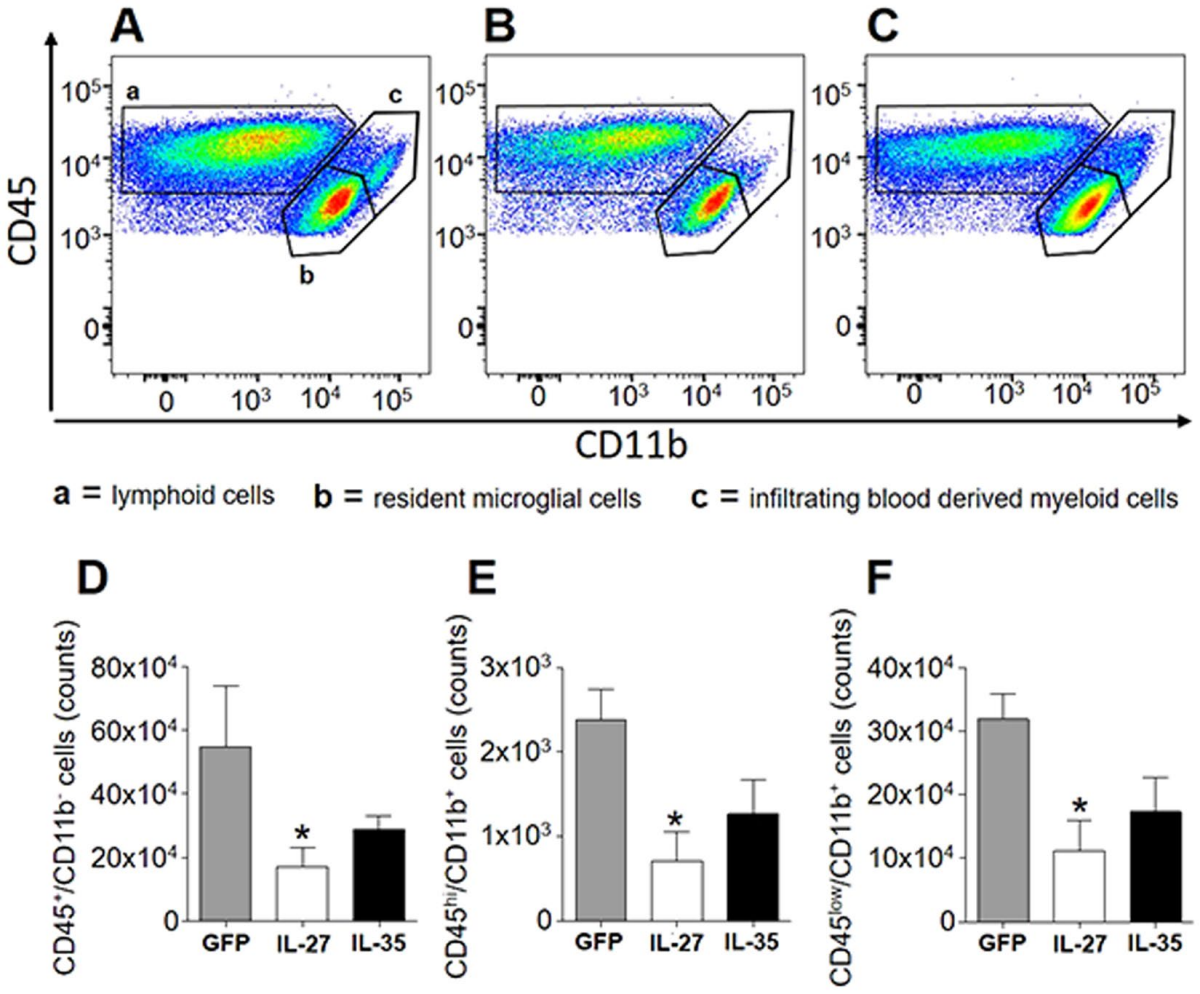
IL-27HA and IL35HA modulate the number of microglia and CNS infiltrating lymphocytes and macrophages. FACS analysis of CNS-infiltrating cells from Lenti-GFP (**A**), IL-27HA (**B**) and IL-35HA (**C**) treated EAE mice, for total CD45^+^CD11b^−^ lymphoid cells, CD45^hi^CD11b^+^ infiltrating macrophages, and CD45^low^CD11b^+^ resident microglia. Lenti-IL-27HA exhibited ability to reduce the number of CNS-infiltrating lymphocytes (**D**) and infiltrating macrophages (**E**). *p < 0.05; (1 way anova; (**D**–**F**) n = 6/group).

We then induced neuroinflammation in C57BL/6 female mice by immunization with MOG_35-55_. We treated EAE mice either the day after immunization (Fig. 2C,D; Supplementary Fig. 5A–D) or the day of clinical disease onset, corresponding to 12–16 days after immunization for most mice (Fig. 2E,F; Supplementary Fig. 5E–H). IL-27 (Fig. 2C,E; Supplementary Figure 5), but not IL-35 (Fig. 2D,F; Supplementary Figure 5) significantly decreased clinical disease severity, both preventively (Fig. 2C; Supplementary Figure 5A–C) and on established disease (Fig. 2E; Supplementary Figure 5E–G), as compared to Lenti-GFP treated EAE mice. We confirmed, by neuropathological analysis (Fig. 3A–D), a significant protection from demyelination and axonal loss only in Lenti-IL-27HA-treated EAE mice (Fig. 3E,F), while in Lenti-IL-35HA-treated mice only the decrease of inflammatory infiltrates reached statistical significance (Fig. 3G). Lenti-IL-27HA displayed also a significant decrease of CD3^+^ T cells (Fig. 3H). We confirmed by FACS the significant decrease of blood-derived CD45^+^CD11b^−^ (lymphoid cells), CD45^hi^CD11b^+^ cells (myeloid cells), and CD45^low^CD11b^+^ (microglia) in the CNS (Fig. 4D–F) in Lenti-IL-27HA-treated EAE mice.

### IL-27 gene therapy reduces the absolute number of infiltrating GM-CSF^+^ and IL-17^+^ CD4^+^ T cells

**Figure 5:**
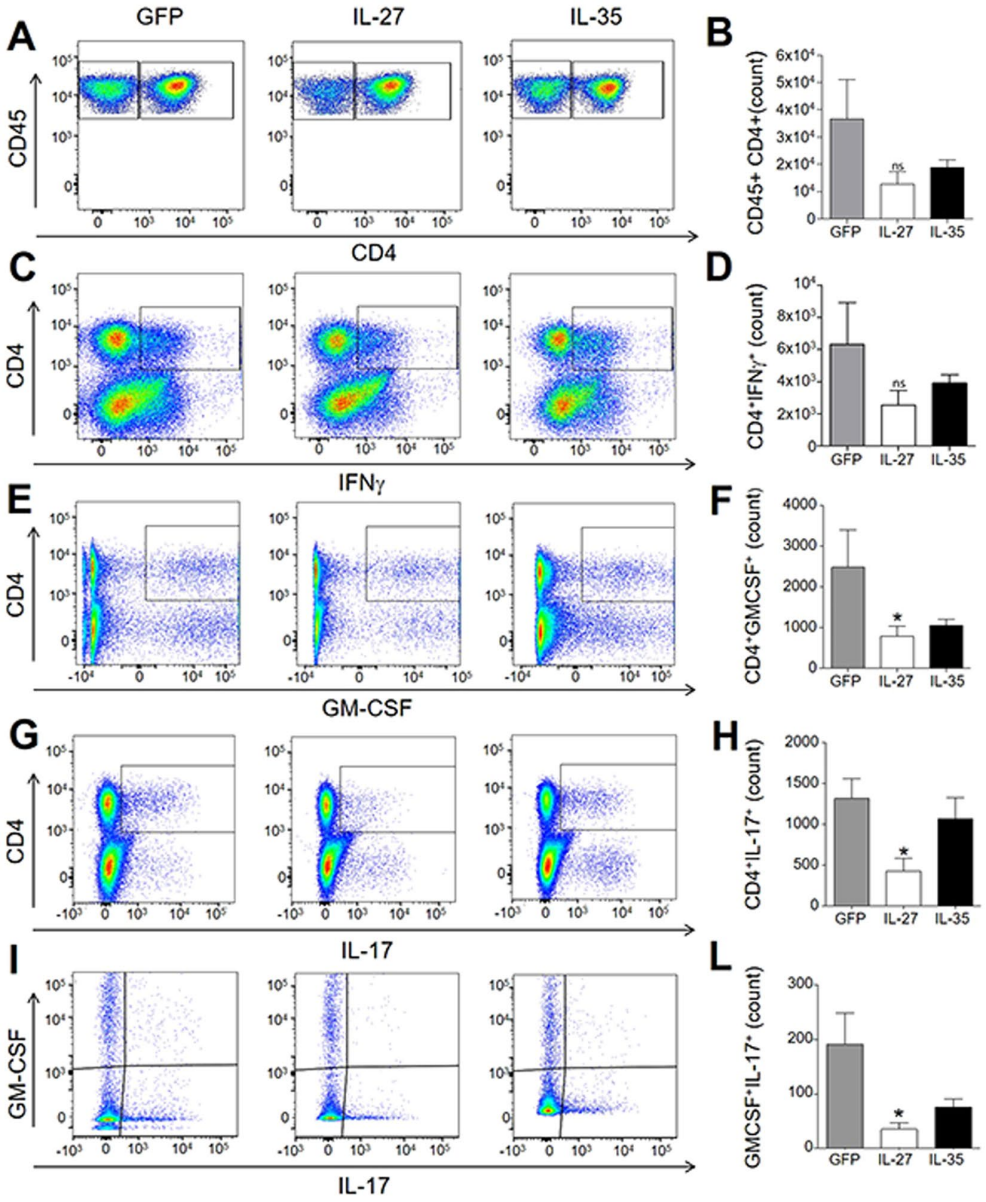
IL-27 gene therapy decreases CNS infiltrating CD4^+^ T cells expressing gm-csf and IL-17. FACS analysis of CNS-infiltrating CD45^+^CD4^+^ T cells isolated from gene therapy-treated EAE mice (**A**,**F**). Intracellular staining for IFNγ (**B**), GM-CSF (**C**), IL-17 (**D**), or GM-CSF/IL-17 (**E**). IL-27, but not IL-35, significantly decreased GM-CSF and IL-17 expression in CD4^+^ T cells (**H**,**I**). Gating strategy is shown in Supplementary Figure. 6. *p < 0.05 (1 way Anova, n = 6/group).

We collected the brains and spinal cords of EAE mice treated with Lenti-IL-27HA and Lenti-IL-35HA and purified infiltrating cells to perform flow cytometric analysis as shown in Supplementary Fig. 4. The decrease of CD45^+^CD4^+^ cells in IL-27-treated EAE mice did not reach statistical significance (Fig. 5A,B), as opposed to the significant decrease in CD4^+^ T cells expressing GM-CSF (Fig. 5E,F), IL-17 (Fig. 5G,H) or both (Fig. 5I,L) in Lenti-IL-27HA-treated but not in Lenti-IL-35HA-treated EAE mice. The latter displayed decreased levels not reaching, however, statistical significance with this sample size (Fig. 5D,F,H,L). Also the decrease of CD4^+^IFNγ^+^ T cells was not significant neither in IL-27, nor in IL-35-treated EAE mice (Fig. 5C,D).

### IL-27, but not IL-35, gene therapy inhibits gm-csf and induces pd-l1 expression in the CNS of EAE mice

**Figure 6:**
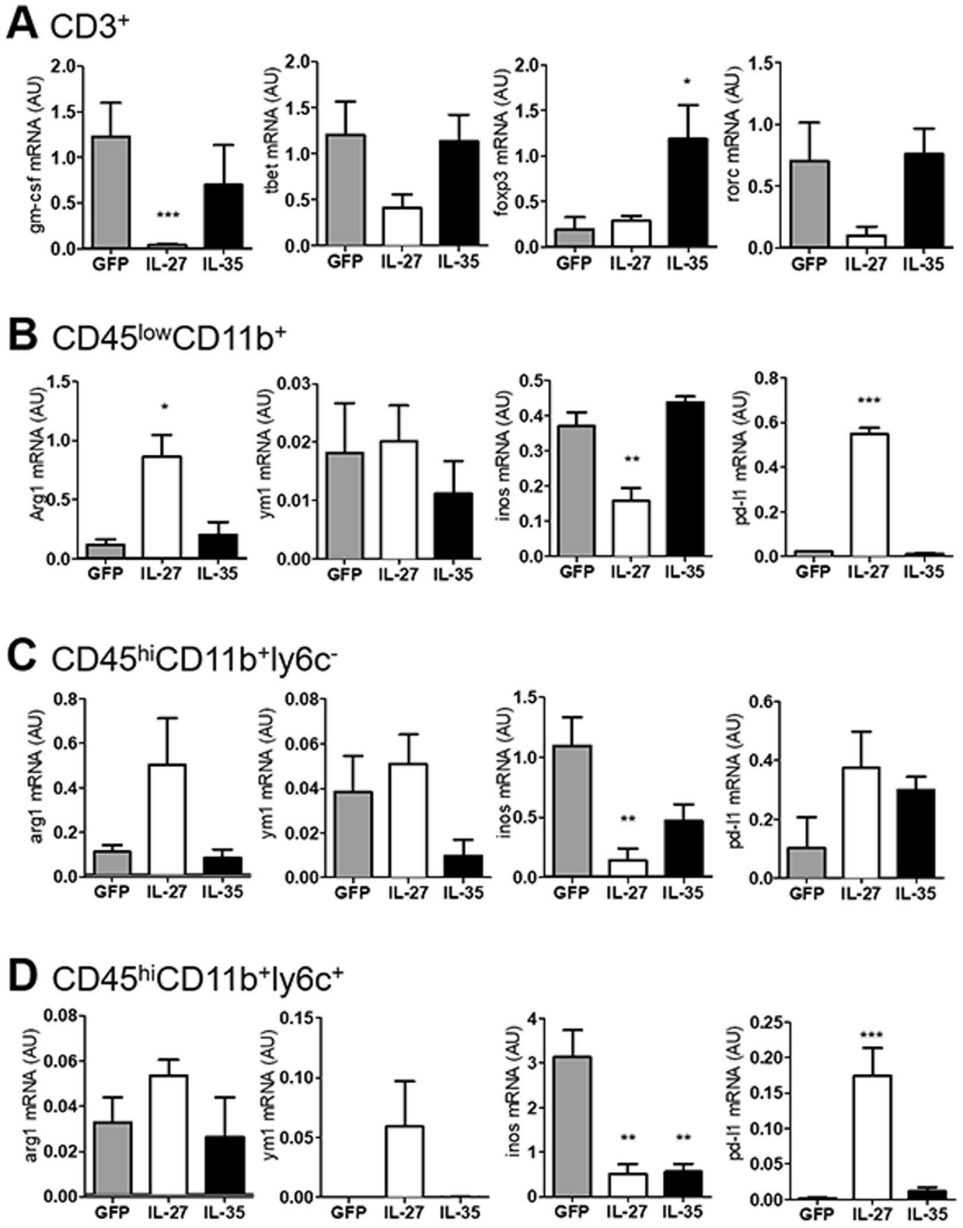
IL-27 and IL-35 gene therapy modulate gene expression in resident microglia and in infiltrating T and myeloid cells. We sorted CNS-resident and -infiltrating cells from gene therapy-treated EAE mice dividing them in T cells (**A**), microglia (**B**), experienced (**C**), and newly infiltrating monocytes/moDCs (**D**), according to the strategy depicted in see Supplementary Fig. 4. Gene expression for gm-csf, tbet, foxp3, rorc (**A**), arg1, ym1, inos, and pd-l1 (**B**–**D**) was measured by real time RT-PCR and expressed as arbitrary units AU (mean ± sd). *p < 0.05, **p < 0.01, ***p < 0.001 (unpaired t-test, n = 6 mice/group).

We next prepared infiltrating cells from treated EAE mice and sorted by flow cytometry (Supplementary Fig. 6), four different populations: CD45^+^CD11b^−^CD3^+^ T cells, CD45^low^CD11b^+^ly6c^−^ cells (putatively microglia), CD45^high^CD11b^+^ly6c^−^ cells (experienced macrophages), and CD45^high^CD11b^+^ly6c^+^ cells (recently infiltrated monocytes, including monocyte-derived dendritic cells). We found, by RT-PCR, that intrathecal IL-27, but not IL-35, gene therapy significantly decreased gm-csf levels in infiltrating T cells, without significantly affecting the mRNA levels of tbet (Th1), foxp3 (Treg), or rorc (Th17) (Fig. 6A). T cells from IL-35-treated mice displayed significantly increased levels of foxp3 (Fig. 6A). IL-27 repressed inos mRNA expression in microglia (Fig. 6B), experienced macrophages (Fig. 6C) and newly infiltrating (Fig. 6D) monocytes, while IL-35 only inhibited inos in the latter (Fig. 6D). IL-27 inconsistently up-regulates the anti-inflammatory markers arginase and chitinase-1 (significant only in microglia, Fig. 6B), but dramatically induces pd-l1 expression in microglia and newly infiltrating monocyes/moDCs (Fig. 6B,D).

## Discussion

IL-27 and IL-35, the two members of the IL-12 family thought to have anti-inflammatory properties, have been investigated in neuroinflammation mainly indirectly through the use of mutant mice devoid of either the subunits forming these heterodimeric cytokines, or their receptors^8,9,16,18,21^. We decided to investigate the effect of IL-27 and IL-35 directly in the CNS, expressing these heterodimeric cytokines from a single polypeptide chain from a lentiviral backbone. The choice to express cytokines in the CNS by injecting viral vectors in the CSF has several advantages, allowing to target multifocal diseases such as EAE and MS, to have prolonged release after a single injection, and to avoid peripheral effects. Using this approach we have successfully investigated the activity of a number of cytokines in the past (i.e. IL-4, IFNγ, IL-1RA, FGF-II, IL-25)^22,23^. The expression of heterodimeric cytokines from a single polypeptide chain poses the problem of their biological activity. We show that IL-27 and IL-35 produced by our lentiviral vectors and purified on affinity columns, work properly *in vitro*. IL-27HA and IL-35HA exhibited activity very similar to commercial recombinant IL-27 and IL-35. Analogue constructs for IL-35 have been successfully used in the past in different experimental settings^13,21^.

We have found that IL-27 and, less consistently, IL-35, decrease in a dose-dependent way GM-CSF, from stimulated CD4^+^ T cells, both at protein and at the mRNA level. The effect of IL-27 on GM-CSF had already been reported *in vitro* in the past^8,11^, but we confirm here by FACS and qRT-PCR on sorted CNS-infiltrating CD3^+^ T cells that the same occurs in EAE. IL-27 may suppress GM-CSF production by Jak2/Tyk2 and Stat1 signaling as reported by Young *et al*.^8^. GM-CSF is one of few cytokines whose genetic deletion confers complete resistance to EAE development^11^. Monoclonal antibody to GM-CSF has been tested for its safety in human multiple sclerosis (MS)^24^ and it is already used in the therapy of rheumatoid arthritis^25^. GM-CSF from CNS-infiltrating T cells is believed to license infiltrating monocytes to tissue damage^12^. IL-27 has shown to modulate GM-CSF production from CD4^+^ T cells, and since GM-CSF serves as communication conduit between tissue-invading lymphocytes and myeloid cells^26^, we wondered if IL-27 affects the recruitment of infiltrating monocytes and influences the reactivity of resident myeloid cells. We found that microglia, macrophages, and newly activated monocytes and monocyte dendritic cells (moDCs), defined byCD45, CD11b, and Ly6c expression^12^, are differently modulated by IL-27 and IL-35. While IL-27-dependent expression of PD-L1 in infiltrating macrophages has been already shown^27^, we describe here, for the first time, that IL-27 is able to dramatically increase pd-l1 mRNA in microglia *in vivo*. We confirmed *in vitro*, on a microglia cell line, that IL-27 up-regulates PD-L1 also at the protein level (data not shown). The ability of IL-27 to decrease iNOS in macrophages and to induce PD-L1 in T cells was previously described *in vitro and in vivo* ^28,29^. IL-27, therefore, may act not only on T cells but also in myeloid cells. Its effect on dendritic cells has been extensively described by others^30^, but very little is known about microglia and monocytes. Moreover, negative modulation of the PD-1/PD-L1 pathway has been crucially linked to MS development and severity^29,31^.

The inhibition of GM-CSF in T-cells, and induction of PD-L1 in myeloid cells, might be one of the mechanisms by which IL-27 but not IL-35, inhibits both developing and established EAE. It has been already reported that the subcutaneous administration of recombinant IL-27 promotes clinical recovery in adoptive, relapsing-remitting, and chronic EAE^8^. This effect was associated mainly to the modulation of Th17 cells infiltrating the CNS and the induction of IL-10^8,9,32,33^. IL-35 gene therapy did not modify disease course, but achieved some degree of tissue protection. The absence of clinical correlate, in the case of IL-35, may be due to the clinical score scale, considering mainly gait, poorly sensitive to small changes. On the other hand clinical signs in EAE are mainly consequence of edema in the spinal cord, and IL-27 might be more efficient than IL-35 in reducing the vasogenic edema^34^. What is known so far about IL-35 in EAE is that IL-35-producing B cells plays negative regulation of immunity, and mice in which only B cells did not express IL-35 lost their ability to recover from EAE^16^. A similar mechanism was reported in mice affected from experimental autoimmune uveitis^21^. We found that IL-35 gene therapy was associated to increased mRNA levels of foxp3 in CNS-infiltrating CD3^+^ T cells. While the up-regulation of IL-35 by FoxP3 has been already reported^13^, the opposite has never been shown before. The pictures that emerges from these data is that IL-27, differently from IL-35, when administered directly into the inflamed brain, may inhibit GM-CSF from CD4^+^ T cells, and induces pd-l1 mRNA in microglia and infiltrating monocytes, with the final result to inhibit EAE development. Our data represent the first *in vivo* demonstration of this protective circuit that might open therapeutic perspectives for neuroinflammatory diseases.

## Materials and Methods

### IL-27HA and IL-35HA lentiviral vector construction

The Ebi3 β and the p35 subunits were cloned from RNA extracted from SJL mice spleen with specific primers:

– Ebi3 Forward primer 5′-CGGGATCCACCATGTCCAAGCTG-3′

– Ebi3 Reverse primer 5′-GGGTCGACGGAGATATCGGAACC-3′

– p35 Forward primer 5′-GGGATATCATGTGTCAATCACG-3′

– p35 Reverse primer 5′-TTCGTCTCAGGTAGGCGGAGCT-3′

The p28 subunit was purchased from Life Technologies.

The coding sequence for the hemagglutinin (HA) epitope tag was inserted in a polylinker by ligation of four oligos, pol1: 5′-ATC AAC CGT CTC ATA CCC ATA CGA CGT GC-3′; pol2: 5′- CAG ACTACG CAT AGG-3′; pol3: 5′-TCG ACC TAT GCG TAG TCT GGC ACG-3′; pol4: 5′-TCG TAT GGG TAT GAG ACG GTT GAT-3. The two subunits were linked by a 12aa linker GGSGGSGGSGGS (GGSlinker) as shown in Fig. 1. Coding sequences of IL-27HA and IL-35HA were extracted from pBSIISK plasmids, using BamHI and SalI enzymes, and cloned into p277 lentiviral transfer vector. Lentiviruses expressing mIL-27HA (Lenti-IL-27HA) and IL-35HA (Lenti-IL-35HA) were generated as previously described. As negative control, a GFP-expressing lentivirus (Lenti-GFP) was also used in all experiments.

### IL-27HA and IL-35HA purification

HEK293T cells were grown in IMDM supplemented with 10% of fetal bovine serum (FBS), 2 mM ultra-glutamin and 100 U ml penicillin/streptomycin (Lonza, Braine-lAlleud, Belgium). Cells were seeded at 8 × 10^6^ in a 15 cm petri disch (Corning Incorporated Life Sciences, Lowell, MA, USA) and, 24 h later, infected with Lenti-IL-27HA, Lenti-IL-35HA, or Lenti-GFP. After 48 h, IL-27HA, IL-35HA conditioned medium were collected for HA affinity column purification. 50 mL supernatant from IL-27HA- or IL-35HA-expressing cells were centrifuged at room temperature for 15 minutes at 20.000 × g and then passed through a 0.22 µm filter. Clarified supernatants were incubated at RT over-night with 0.2 mL of anti-HA antibody agarose resin (cat. A2095, Sigma Aldrich) using an Amicon Pro centrifuge purification system (Merck Millipore). After three washes with PBS, bound material was eluted by applying 1 mL of 0.1 mg/mL HA peptide (cat. I2149, Sigma Aldrich) in PBS. Purified proteins were stored at −80 °C.

### CD4^+^ T cells polarization assay

Duplicate cultures of CD4^+^ cells/well in 200 μl of RPMI were isolated from the spleens of C57Bl/6 N mice. CD4^+^ T cells were purified using anti-CD4 beads (Milteny). Polarization was performed by stimulating with 5 µg/mL of plated-bound anti-CD3 and 5 µg/mL soluble anti-CD28 (BD Biosciences, Mountain View, CA) for 72 h. Stimulation of T cells was set up under optimal culture conditions with or without recombinant cytokines and/or neutralizing antibodies. Th0: anti-CD3/anti-CD28; Th1: anti-CD3/anti-CD28, anti-IL4 (5 µg/mL), mouse IL-12 (10 ng/mL); Th17: anti-CD3/anti-CD28, mouse IL-6 (30 ng/mL), human tgf-β (3 ng/mL). After 24 h, polarized T cells were treated with purified IL-27HA, IL-35HA, and recombinant IL-27 (R&D system) or recombinant IL-35 (Sigma Aldrich) at 10 and 50 ng/mL. IL-27HA concentration was determined using the commercial ELISA described below. IL-35HA concentration was estimated by WB using IL-27HA as comparison. After 48 h of cytokine stimulation, T cells were collected for RNA extraction and supernatants for cytokine determination.

### RT-PCR

Total RNA was extracted from CD4^+^ T cells and from CNS infiltrated cells of the EAE mice with RNeasy Mini Kit (Qiagen). Genomic DNA was removed by treatment with DNAse I type (Qiagen). cDNA synthesis was performed using Thermoscript^TM^ RT-PCR system (Invitrogen). Arg-1 (Mm00475988_m1); inos (Mm00440502_m1); ym1 (Mm00657889_mH); cd274 (Mm00452054_m1); tbx21 (Mm00450960_m1); csf2 (Mm01290062_m1); IL-17a (Mm00439618_m1); rorc (Mm00441144_g1); foxp3 (Mm00475156_m1); IL-2 (Mm00434256_m1); IL-10 (Mm00439614_m1) and gapdh (4352339E) mRNA levels were measured by real-time RT-PCR (Applied Biosystem, Invitrogen). The 2^−ΔΔCT^ method was used to calculate relative changes in gene expression^35^.

### Isolation of CNS infiltrating leukocytes

Extracted brain and spinal cord tissues were incubated for 30 minutes with 0.4 mg/mL type IV collagenase (Sigma-Aldrich) and dissociated using a 19 G syringe to obtain a homogenous cell suspension. Finally, CNS cells were enriched by a Percoll gradient as previously described^36^.

### Flow cytometry and sorting

Flow cytometry was performed using a CantoII (Becton Dickinson) and analysed with FlowJo software (tree star). Fluorochrome-conjugates monoclonal antibody (mABs) specific for CD45 (clone 30-F11), CD11b (clone M1/70), CD4 (clone RM4-5), CD3 (17A2), Ly6C (clone AL-21), Ly6G (clone 1A8), CCR2 (clone #47503) were purchased either from BD Biosciences, eBioscience, R&D and Biolegend.

For intracellular staining, cells were stimulated for 4 h with phorbol 12-myristate 13-acetate (50 ng/ml) and ionomycin (500 ng/ml) in the presence of GolgiPlug (1:1000, BD Pharmigen), permeabylized using a Cytofix/Cytoperm Plus kit (BD Bioscience) and stained with the following fluochrome-conjugates monoclonal antibodies: GM-CSF (clone MP1-22E9), IL-17A (TC11-18H10) and IFNγ (clone XMG1.2) from BD Pharmingen. Dead cells were excluded using a Zombie Nir, Biolegend.

### ELISA assay for mIL-27

Mouse IL-27 was measured in supernatants from the HA purification assay, in conditioned media and in CSF, withdrawn from mice cisterna magna by capillarity, of Lenti-IL-27HA-injected EAE mice, using a DUOset ELISA for mouse IL-27 (R&D Systems).

### Western blot analysis

30 μg of proteins or 10 μl of Lenti-IL-27HA and Lenti-IL-35HA treated-mice CSF were subjected to SDS-12% polyacrylamide gel electrophoresis before blotting onto a PVDF membrane, PROTRAN Nitrocellulose Transfer Membrane (Cat. No. 8160576 WHATMAN). Membranes were blocked with 5% skimmed milk in Tris-buffered saline with 0.05% Tween 20 for 1 h at RT, and were probed with Purified rabbit anti-HA tag polyclonal (ab175880) O/N at 4 °C. A suitable secondary peroxidase-conjugated antibody was used for detection using ECL Immobilon Western Chemiluminescent HRP-substrate detection system (Millipore, Cat.No. WBK 250500) according to manufacturer’s instructions.

### Mice

All procedures involving animals were performed according to the animal protocol guidelines prescribed by the Institutional Animal Care and Use Committee (IACUC) authorization no. 644 at San Raffaele Scientific Institute (Milan, Italy).

Six- to eight-weeks-old C57Bl/6 female mice were purchased from Charles River Laboratories (Calco, Italy). All mice were housed in specific pathogen-free conditions, in roomy cages, allowing free access to food and water. All efforts were made to minimize animal suffering and to reduce the number of mice used, in accordance with the European Communities Council Directive of November 24, 1986 (86/609/EEC).

### IL-27HA and IL-35HA gene therapy

Chronic EAE was induced in female C57BL/6 mice by subcutaneous immunization with 300 μl of 200 μg per mouse of MOG_35–55_ in Freund’s Adjuvant Incomplete liquid, IFA, (Sigma) supplemented with 8 mg ml−1 Mycobacterium tuberculosis (strain H37Ra; Difco, Lawrence, KS, USA). Pertussis toxin (500 ng, List Biological Laboratories, Campbell, CA, USA) was injected i.v. on the day of the immunization and again 2 days later. IL-27HA and IL-35HA-expressing lentivirus or GFP-expressing lentivirus were injected in the cisterna magna (i.c of the mice) in preventive, one day post immunization, or in therapeutic, disease onset^20^. Mice were weighed and scored for clinical signs daily up to the day of culling. Clinical assessment of EAE was performed according to the following scoring criteria: 0 = healthy; 1 = limp tail; 2 = ataxia and/or paresis of hindlimbs; 3 = paralysis of hindlimbs and/or paresis of forelimbs; 4 = tetraparalysis; and 5 = moribund or death. EAE mice were killed at 28–34 d.p.i for real-time PCR, histological, and FACS analysis.

### Histological evaluation

At least five mice per group were perfused through the left cardiac ventricle with saline plus EDTA 0.5 mM for 10 min followed by fixation with cold 4% paraformaldehyde, PFA, (Sigma). Spinal cords and brains from EAE mice were dissected out and post-fixed in 4% PFA overnight. Four different staining were used: Hematoxylin and Eosin to detect inflammatory infiltrates, demyelination (Kluver Barrera), axonal damage (Bielshowsky), and immunohistochemistry for CNS infiltrating CD3+ T (MCA1477, Serotec). Findings were quantified on an average of 10 complete cross-sections of spinal cord per mouse taken at eight different levels. The number of perivascular inflammatory infiltrates were calculated and expressed as the number of inflammatory infiltrates per mm^2^, demyelinated areas and axonal loss were expressed as percentage of damaged area, and CD3+ T cells were calculated and expressed as the number of cells per square millimetre.

### Statistical analysis

Statistical evaluations were expressed as mean ± s.d. or mean ± s.e.m, as appropriate. Results were analyzed using one way anova, unpaired Student’s t-test and Mann–Whitney U-test for samples with unknown and potentially disparate variances. Statistical significance was ranked *p < 0.05, **p < 0.01, ***p < 0.001.

## Electronic supplementary material

Supplementary Information
