# Supplementary material for: IL-27, but not IL-35, inhibits neuroinflammation through modulating GM-CSF expression
Source: Sci Rep. 2017 Nov 29;7:16547. doi: 10.1038/s41598-017-16702-w (PMC5707351; doi:10.1038/s41598-017-16702-w)

SUPPLEMENTARY INFORMATION

i. Supplementary Methods

ii. Supplementary Figure 1-7

IL-27, but not IL-35, inhibits neuroinflammation through modulating GM-CSF expression

Giacomo Casella[1], Annamaria Finardi[1], Hélène Descamps[2], Federico Colombo[1], Chiara Maiorino[1], Francesca Ruffini[2], Marco Patrone[3], Massimo Degano[3], Gianvito Martino[2], Luca Muzio[2], Burkhard Becher[4], and Roberto Furlan[1*]

[1]Clinical Neuroimmunology Unit, Department of Neuroscience, Institute for Experimental Neurology, San Raffaele Scientific Institute, 20132 Milan, Italy

[2]Neuroimmunology Unit, Department of Neuroscience, Institute for Experimental Neurology, San Raffaele Scientific Institute, 20132 Milan, Italy

[3]Biocrystallography Unit, Department of Immunology, Transplantation and Infectious Diseases, San Raffaele Scientific Institute, 20132 Milano, Italy.

[4]Inflammation Research, Institute of Experimental Immunology, University of Zurich, 8057 Zurich, Switzerland.

*Address correspondence to: Dr. Roberto Furlan, Clinical Neuroimmunology Unit, Institute of Experimental Neurology (InSpe), San Raffaele Scientific Institute, Via Olgettina 58, 20132 Milan, Italy. Email address: furlan.roberto@hsr.it

Supplementary Methods

Multiplex cytokine assay

Supernatants from $CD4^+$ T cell culture experiments were collected and cytokines concentration was analyzed with Bio-plex Pro^{TM} Mouse Cytokines Standard 23-Plex, Group I (Biorad) according to the manufacturer's instructions. Samples were analyzed in duplicate and read by Luminex (Biorad). Bio-Plex manager 4.0 software was used for the data analysis.

MTT assay

Cytotoxicity was assessed by MTT assay in 96-well plates. Briefly, after the exposure to 10ng or 50 ng of IL-27 or IL-35 for 72h at 37 C, the cell cultures were incubated with 5 mg/ml MTT solution (Sigma, Milano, Italy). After 2h at 37°C, cells were pelleted by centrifugation and formazan blue precipitates were dissolved by DMSO under gentle agitation on an orbital shaker. Absorbance values were determined at at 570 nm; the O.D. of untreated cells was used as reference and expressed as 100% of cell viability.

CFSE proliferation assay

$CD4^+$ T cells were isolated from a spleen of C57 wild type mice, using microbeads (Milteny). The cells were labeled with CFSE (Thermofisher), activated with anti-CD3 antibody (5µg/mL) and anti-CD28 (5µg/mL). After 24 h, T cells were treated with purified IL-27HA and IL-35HA at 10 and 50 ng/mL. On day 4, the cells were stained with PB-conjugated anti-CD4 (BD Pharmingen). Dilution of CFSE in $CD4^+$ T cells was measured by flow cytometry.

Supplementary Figures

Supplementary Figure 1. CD4 polarization, proliferation, and viability. $CD4^+$ polarization was confirmed by RT-PCR and Multiplex cytokines assay. Tbx21 and rorγ mRNA, IFNγ and TNFα were analyzed in Th0, Th1, and Th17 polarized cells (**A-D**). IL-27HA and IL35-HA toxicity was measured by MTT assay. $CD4^+$ T cells were left unstimulated (NC), or treated with purified IL-27HA (black dots), or IL-35HA (black triangles) at 10 or 50 ng/ml for 48h (**E**). Cell viability after cytokine treatment was expressed as percentage of untreated cells viability. $CD4^+$ T cells were labeled with CFSE (1.5µM) and incubated with 10 or 50 ng of IL-27HA, and IL-35HA (**F**) for 4 days. Percentages of proliferating cells in each condition (**G**) were evaluated. Control cultures contained no cytokines (NC) or were left unstained.

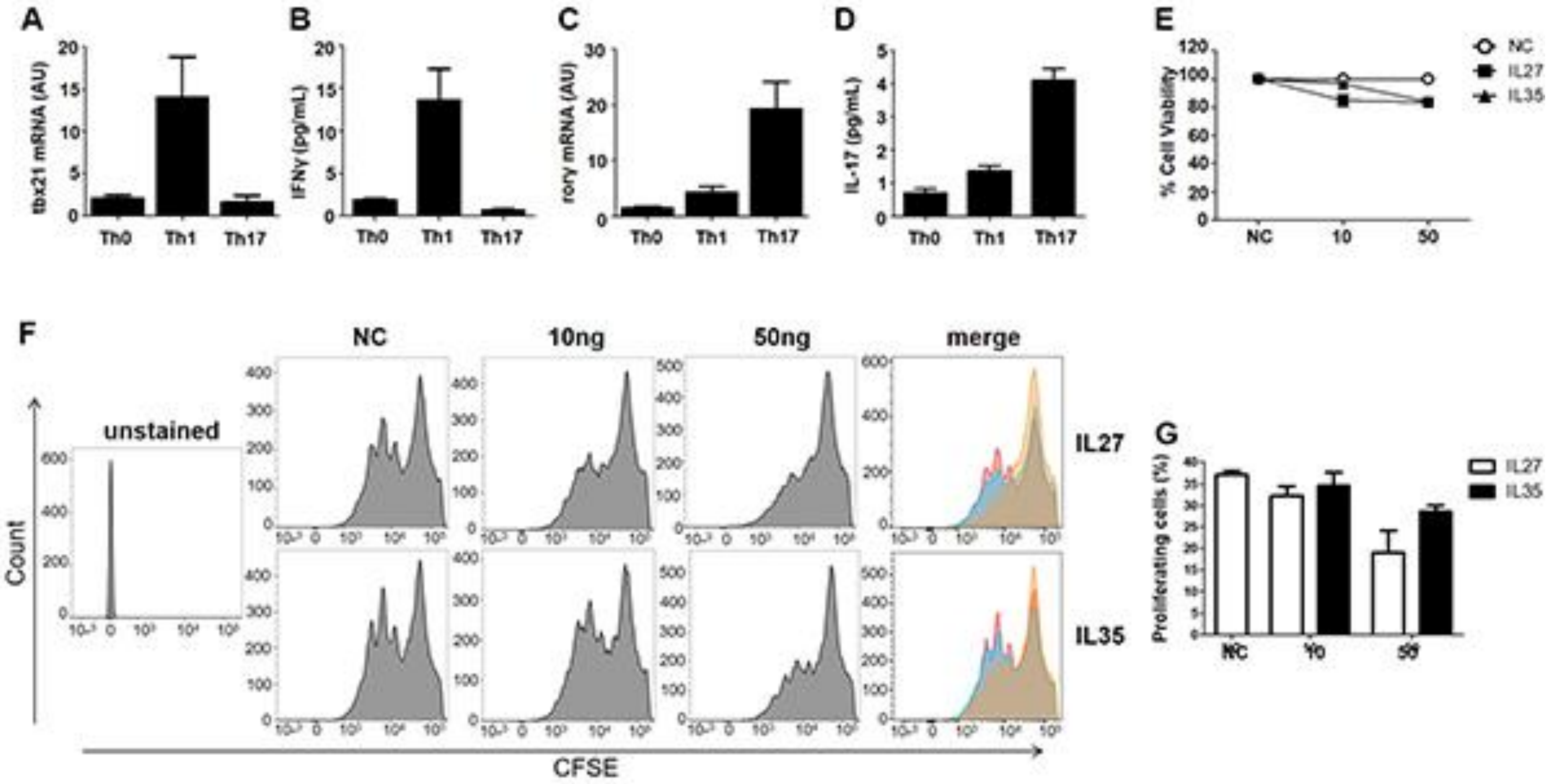
A
tbx21 mRNA (AU)
Th0
Th1
Th17
B
IFNγ (pg/mL)
C
rorγ mRNA (AU)
D
IL-17 (pg/mL)
E
% Cell Viability
NC
IL27
IL35
10
50
F
unstained
NC
10ng
50ng
merge
IL27
IL35
Count
CFSE
G
Proliferating cells (%)

Supplementary Figure 2. Validation of IL-27HA and IL-35HA purified proteins. No cytokines (NC, grey bars), or purified IL-27HA (white bars), or IL-35HA (black bars) were added at 10 or 50 ng/ml for 48h. IL-2 (**A**), GM-CSF (**B**), IL-17 (**C**), IFNγ (**D**), and TNFα (**E**) protein levels were quantified from CD4^{+} Th0 or from polarized Th1, Th17 cells, *in vitro* (mean ± sd). *= p<0.05, **=p<0.001, ***=p<0.0001 (1 way anova).

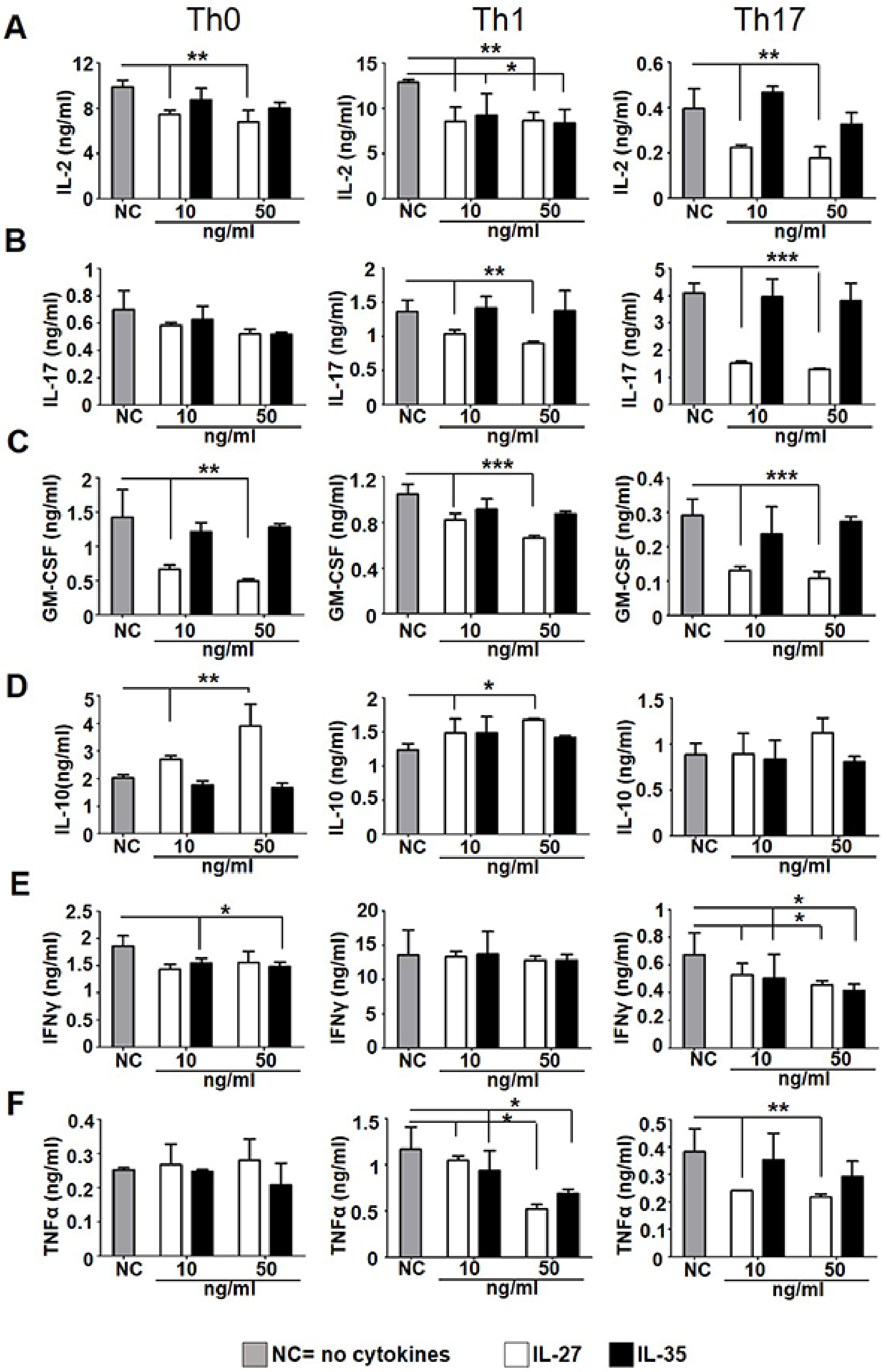

Supplementary Figure 3. Comparison of IL-27HA activity with commercial IL-27. No cytokines (NC, grey bars), or purified IL-27HA (white bars), or commercial rIL-27 (black bars) were added at 10 or 50 ng/ml for 48h. IL-2 (**A**), IL-17 (**B**), GM-CSF (**C**), IL-10 (**D**), IFNγ (**E**), and TNFα (**F**) protein levels were quantified from CD4^{+} Th0 or from polarized Th1, Th17 cells, *in vitro* (mean ± sd). *= p<0.05, **=p<0.001, ***=p<0.0001 (1 way anova).

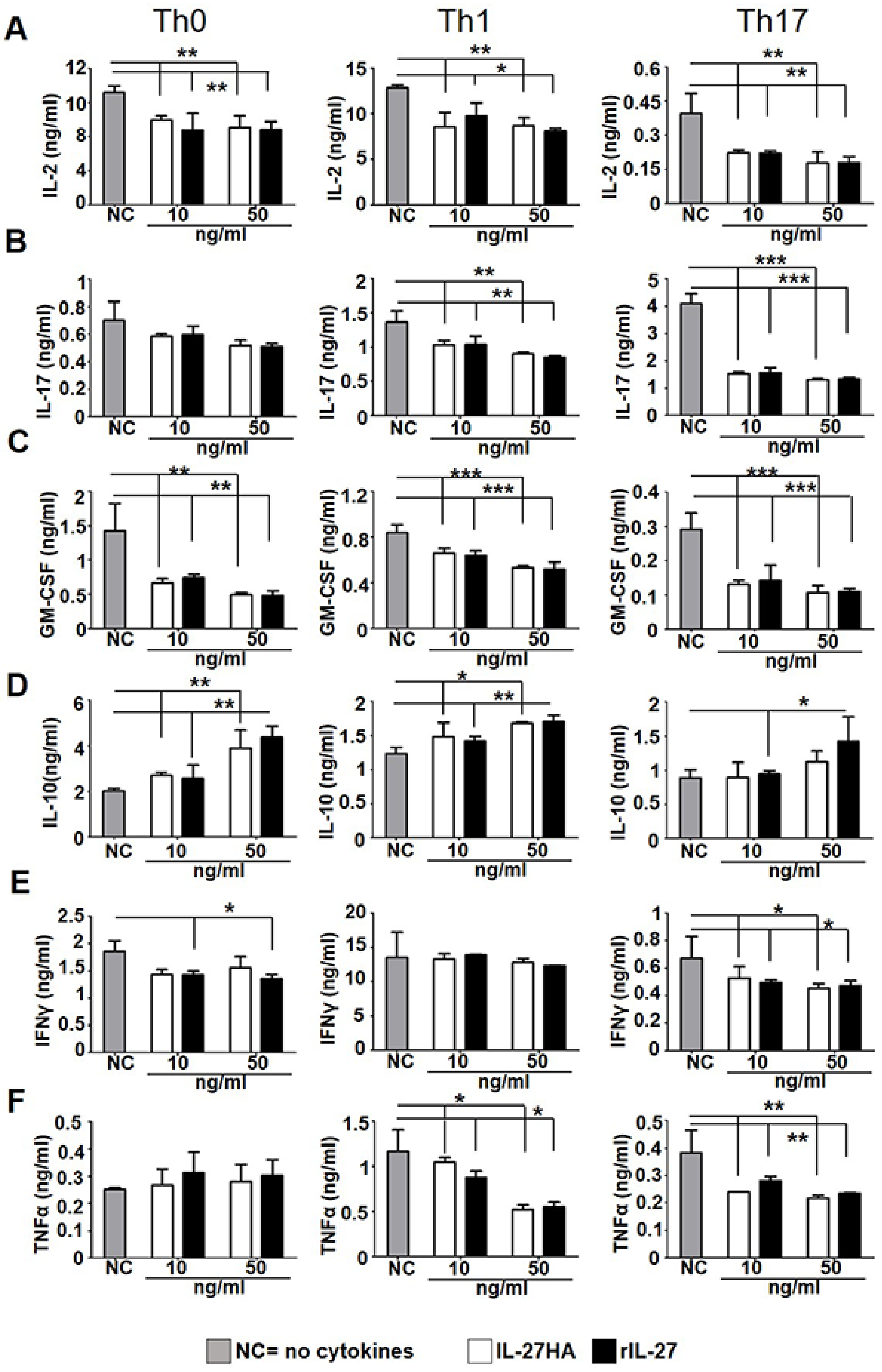

Supplementary Figure 4. Comparison of IL-35HA activity with commercial IL-35. No cytokines (NC, grey bars), or purified IL-35HA (white bars), or commercial rIL-35 (black bars) were added at 10 or 50 ng/ml for 48h. IL-2 (**A**), IL-17 (**B**), GM-CSF (**C**), IL-10 (**D**), IFNγ (**E**), and TNFα (**F**) protein levels were quantified from CD4^{+} Th0 or from polarized Th1, Th17 cells, *in vitro* (mean ± sd). *= p<0.05, **=p<0.001 (1 way anova).

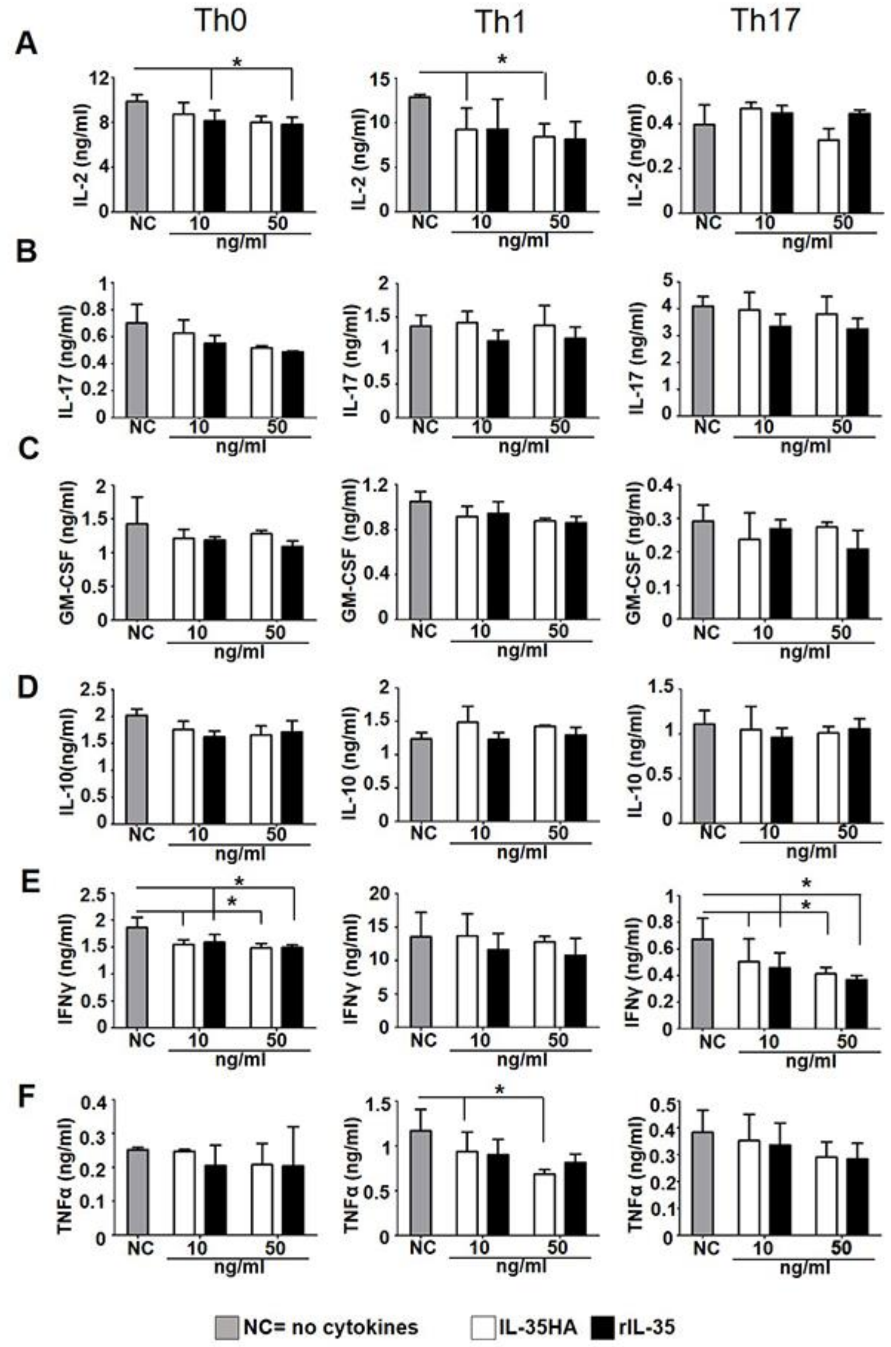

Supplementary Figure 5. IL-27, but not IL-35 gene therapy inhibits clinical EAE development. Mean clinical score of other two EAE experiments. The mice were injected with Lenti-IL-27HA and Lenti-IL-35HA (open dots) or Lenti-GFP (closed dots) both preventively (**A-D**), on the day after immunization (1 d.p.i), and therapeutically, on the day of disease onset (11-13 d.p.i) (**E-H**). The mice were sacrificed between 28-34 days post immunization, in according with our experimental strategy. *= p<0.05; **=p<0.001 (EAE is evaluated as cumulative score using Mann Whitney; n= 10 each group in **A-D**, **G-H**; n= 8 each group in **E-F**).

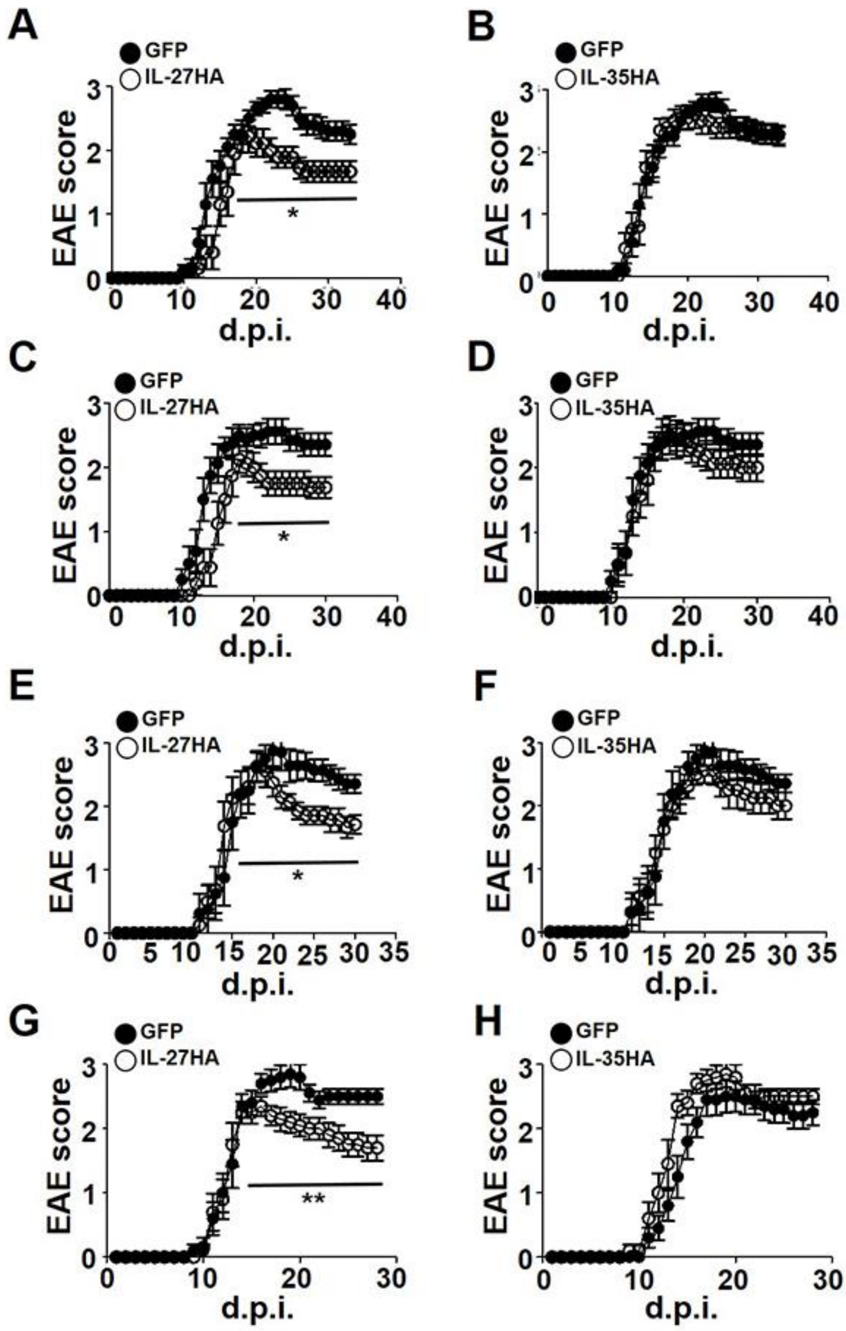

Supplementary Figure 6. FACS gating strategy for the analysis and sorting of CNS-infiltrating cells from EAE mice. Panel **A** shows the gating strategy to identify CNS-infiltrating $CD4^+$ T cells analyzed by intracellular cytokine staining in Figure 5, while panel **B** shows the gating strategy to define the four population sorted and analyzed for gene expression in Fig. 6.

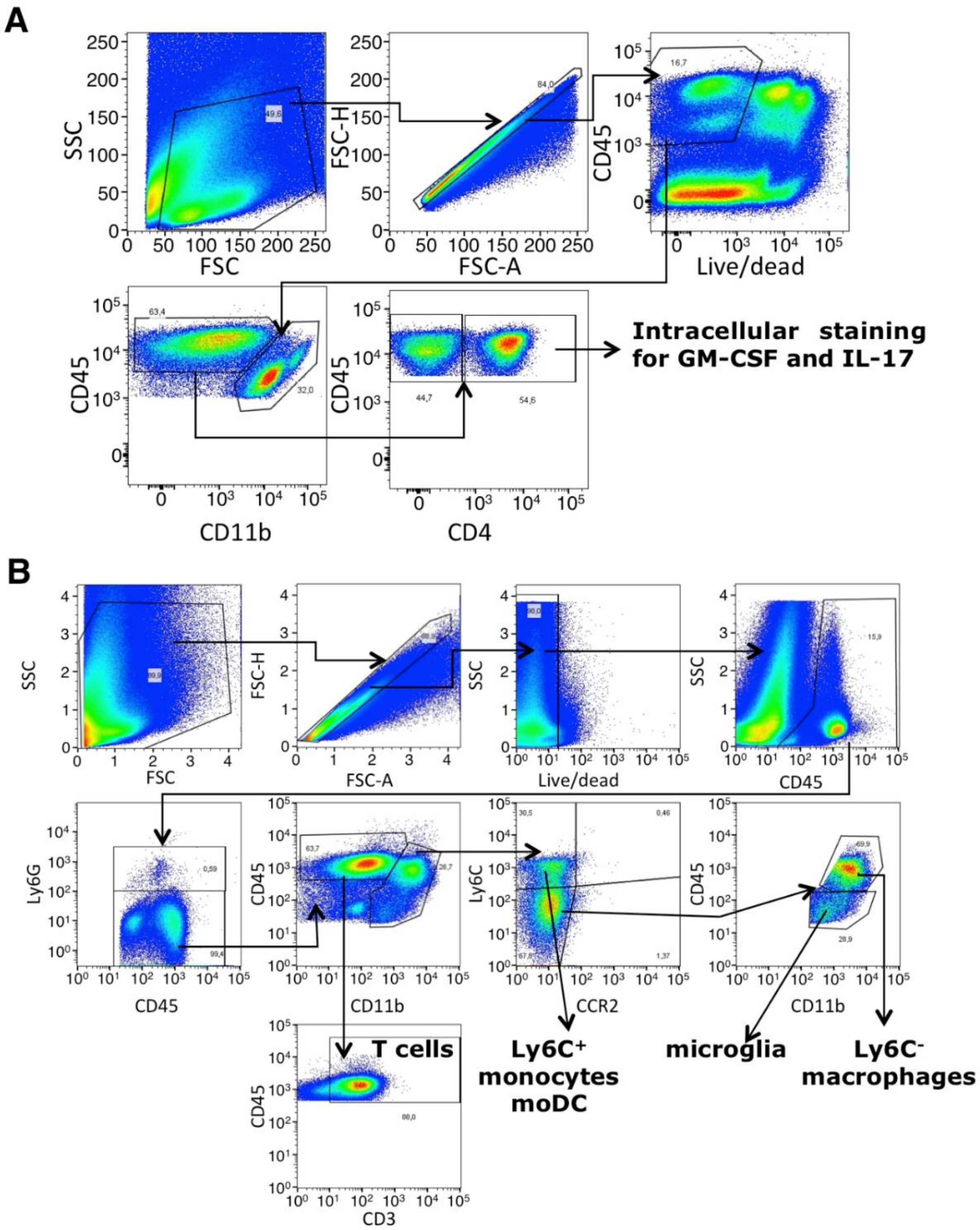

Supplementary Figure 7. Full blots. (**A**) Full blot of cropped blot shown in Fig. 1D. (**B**) Full blot of cropped blot shown in Fig. 2B.

(**A**)

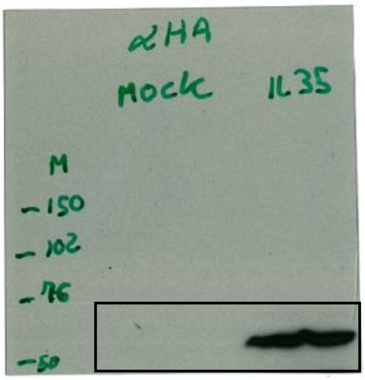

(**B**)

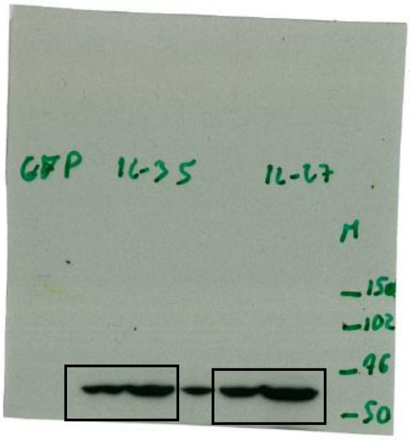

Supplement: Supplementary file 1 — Supplementary Information [file 41598_2017_16702_MOESM1_ESM.pdf]
